# The mitogenomes of two saprophytic Boletales species (*Coniophora*) reveals intron dynamics and accumulation of plasmid-derived and non-conserved genes

**DOI:** 10.1016/j.csbj.2020.12.041

**Published:** 2020-12-30

**Authors:** Peng Wu, Zhijie Bao, Wenying Tu, Lijiao Li, Chuan Xiong, Xin Jin, Ping Li, Mingying Gui, Wenli Huang, Qiang Li

**Affiliations:** a Yunnan Plateau Characteristic Agricultural Industry Research Institute, Yunan Agricultural University, Kunming, Yunnan, China; b School of Food and Biological Engineering, Chengdu University, Chengdu, Sichuan, China; c Biotechnology and Nuclear Technology Research Institute, Sichuan Academy of Agricultural Sciences, Chengdu, Sichuan, China

**Keywords:** Boletales, Mitochondrial genome, Repeat, Gene rearrangement, Evolution, Phylogenetic analysis

## Abstract

The order Boletales is a group of fungi with complex life styles, which include saprophytic and ectomycorrhizal mushroom-forming fungi. In the present study, the complete mitogenomes of two saprophytic Boletales species, *Coniophora olivacea*, and *C. puteana*, were assembled and compared with mitogenomes of ectomycorrhizal Boletales. Both mitogenomes comprised circular DNA molecules with sizes of 78,350 bp and 79,655 bp, respectively. Comparative mitogenomic analysis indicated that the two saprophytic Boletales species contained more plasmid-derived (7 on average) and unknown functional genes (12 on average) than the four ectomycorrhizal Boletales species previously reported. In addition, the core protein coding genes, *nad2* and *rps3*, were found to be subjected to positive selection pressure between some Boletales species. Frequent intron gain/loss events were detected in Boletales and Basidiomycetes, and several novel intron classes were found in two *Coniophora* species. A total of 33 introns were detected in *C. olivacea*, and most were found to have undergone contraction in the *C. olivacea* mitogenome. Mitochondrial genes of *Coniophora* species were found to have undergone large-scale gene rearrangements, and the accumulation of intra-genomic repeats in the mitogenome was considered as one of the main contributing factors. Based on combined mitochondrial gene sets, we obtained a well-supported phylogenetic tree for 76 Basidiomycetes, demonstrating the utility of mitochondrial gene analysis for inferring Basidiomycetes phylogeny. The study served as the first report on the mitogenomes of the family Coniophorineae, which will help to understand the origin and evolution patterns of Boletales species with complex lifestyles.

## Graphical abstract

**Figure:**
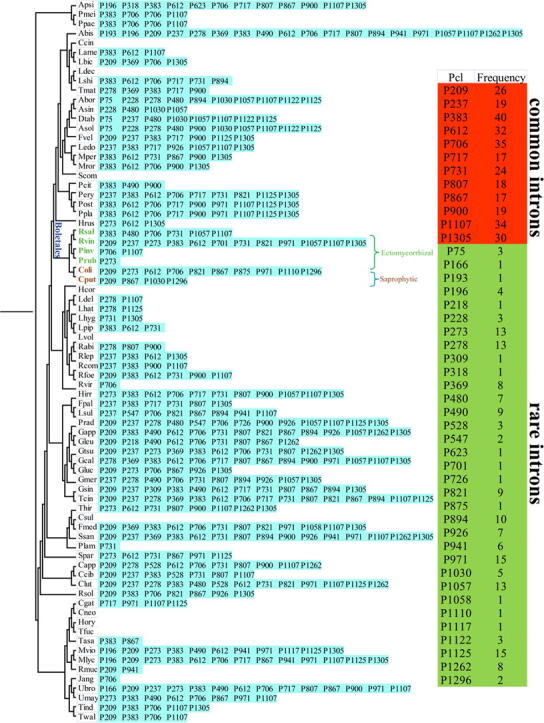

## 1 Introduction

The genus *Coniophora*, which belongs to Boletales, Basidiomycota, is a group of brown-rot fungi [1]. The *Coniophora* species can be found both in building materials and the natural environment in temperate and boreal regions. The *Coniophora* species is characterized by producing brown-rot decay on dead wood of conifers (softwood) in Boletales, which only attack cell wall carbohydrates and leave lignin undigested [2], [3], [4]. Boletales species have diverse lifestyles, including saprophytic and ectomycorrhizal fungal species [5]. It is predicted that the common ancestor of Boletales appeared 84 million years ago (presumably a brown-rot saprotroph), and then differentiated into saprophytic and ectomycorrhizal Boletales species [6], [7]. Genomic sequencing showed that Boletales species with different life styles had varied genomic characteristics, such as genome size, gene content and plant cell wall-degradeing enzyme coding genes (PCWDE) [2], [5], [6]. However, differentiations of mitogenomes between Boletales species with different lifestyles have not been known. Previous studies found that ectomycorrhizal *Amanita* species had more repetitive sequences and fewer intergenic sequences than asymbiotic *Amanita* species in their mitogenomes [8]. Up to now, the mitogenomes of four ectomycorrhizal Boletales species have been reported, including *Rhizopogon salebrosus*, *R. vinicolor* [9], *Paxillus involutus*, and *P. rubicundulus* [10]. However, the mitogenomic characteristics of saprophytic Boletales species and variations between saprophytic and ectomycorrhizal Boletales species have not been revealed.

Variations or mutations in mitogenomes could significantly affect the growth, development and metabolism of eukaryotes [11], [12], [13]. In addition, the mitogenome has been widely used as an effective tool to analyze the origin, classification and phylogeny of species due to its rapid evolution rate, single parent inheritance and several available molecular markers [14], [15]. The phylum Basidiomycota, the largest group of mushroom-forming fungi on the earth, plays an important role in the natural circulation of forest ecosystems and people's production and life [16], [17]. The information inferred from mitogenome analyses promoted better understanding of the origin and population genetics of Basidiomycota [18], [19]. It has been reported that the mitogenomes of Basidiomycota vary greatly in size, genome structure, gene content, gene arrangement, repeat sequence content and intron type [20], [21], [22], [23]. So far, there are less than 120 complete mitogenomes available in public database (https://www.ncbi.nlm.nih.gov/genome/browse#!/overview/), far less than the number of Basidiomycete nuclear genomes available. Boletales is an important group of Basidiomycota, which contains more than 1300 described species with diverse lifestyles. It is one of the model groups to study the lifestyle and environmental adaptive evolution of Basidiomycota. However, up to now, only four mitogenomes from the order Boletales are available, and no saprophytic Boletales mitogenome has been completely assembled.

In the present study, the mitogenomes of two saprophytic Boletales species, including *Coniophora olivacea*, and *C. puteana*, were assembled, annotated, and compared with other ectomycorrhizal Boletales species. The aims of this study are: 1) to reveal the mitogenome characterizations of two Boletales species with saprophytic lifestyles; 2) to reveal the variations or conservations between saprophytic and ectomycorrhizal Boletales species by comparative mitogenomic analysis; 3) to reveal the intron dynamics of *cox1* genes in Boletales and other Basidiomycota mitogenomes; 4) to understand the phylogenetic position and origin of Boletales in the phylum Basidiomycota based on mitochondrial genes. This study served as the first report on mitogenomes of saprophytic Boletales species, which will promote the understanding of the origin, evolution and genetics of the order Boletales.

## 2 Materials and methods

### 2.1 Assembly and annotation of *Coniophora* mitogenomes

We downloaded the raw sequencing data of *C. olivacea* and *C. puteana* from the Sequence Read Archive (SRA) database (acc. SRR4171236 and SRR3927427) [2], [5]. A series of quality control steps were conducted to generate clean reads from the raw sequencing data, which included filtering low-quality sequences by AdapterRemoval v 2 [24] and removing adapter reads by ngsShoRT [25]. We filtered out adaptors and reads with N bases exceeding 10% in the raw reads. And then we discarded reads that contained more than 50% of the low-quality bases (phred quality score ≤ 5). The SPAdes 3.9.0 software was used to assemble mitogenomes with default parameters [26]. There were 2 (with an average size of 38.30 kb) and 3 contigs (with an average size of 26.32 kb) obtained in the SPAdes assembly of *C. olivacea* and *C. puteana* mitogenomes, respecitively. Then, we filled gaps between these contigs by the MITObim V1.9 [27], using the mitogenome of *R. salebrosus* [9] as the reference. In addition, NOVO Plasty [28] was also used to verify the assembly . The obtained complete mitogenomes of *C. olivacea* and *C. puteana* were annotated according to our previously described methods [22], [29]. Briefly, the protein-coding genes (PCGs), rRNA genes, tRNA genes, and introns in the two *Coniophora* mitogenomes were initially annotated using MFannot [30] and MITOS [31], both based on the genetic code 4. Then PCGs were modified or predicted using the NCBI Open Reading Frame Finder [32], and further annotated by BLASTP searches (e-value less than 10^-10^) against the NCBI non-redundant protein sequence database [33]. We detected the intron–exon borders of PCGs by the exonerate v2.2 software [34] using closely related species as the reference. We also predicted tRNA genes in the two *Coniophora* mitogenomes using tRNAscan-SE v1.3.1 [35]. The secondary structures of tRNAs were detected using MITOS with default parameters [31]. rRNA genes were also modified using mitogenomes from the order Boletales as references [9], [10]. Graphical maps of the two *Coniophora* mitogenomes were drawn using OGDraw v1.2 [36].

### 2.2 Sequence analysis of *Coniophora* mitogenomes

Base compositions of the two *Coniophora* mitogenomes and other Boletales mitogenomes were calculated using the DNASTAR Lasergene v7.1 (http://www.dnastar.com/). Strand asymmetries of the 6 Boletales mitogenomes were assessed based on the following formulas: AT skew = [A - T] / [A + T], and GC skew = [G - C] / [G + C] [37]. The pairwise genetic distances between each pair of the 15 core PCGs (*atp6, atp8, atp9, cob, cox1, cox2, cox3, nad1, nad2, nad3, nad4, nad4L, nad5, nad6,* and *rps3*) in the 6 Boletales mitogenomes were calculated using MEGA v6.06 [38] based on the Kimura-2-parameter (K2P) substitution model. The DnaSP v6.10.01 software [39] was used to calculate synonymous (*Ks*) and nonsynonymous substitution rates (*Ka*) for core PCGs in the 6 Boletales mitogenomes. Gene collinearity analysis of 6 Boletales species was conducted by using Mauve v2.4.0 [40].

### 2.3 Repetitive element analysis

BLASTN searches [41] of the two *Coniophora* mitogenomes against themselves were conducted to identify if there were interspersed repeats or intra-genomic duplications of large fragments throughout the two mitogenomes, using an E-value of <10^−10^ as the threshold. We detected tandem repeats (<10 bp in length) in the two mitogenomes using the Tandem Repeats Finder [42]. To detect if there were any gene fragments that natural transferred between nuclear and mitochondrial genomes of the two *Coniophora* species, we performed BlastN searches of the two *Coniophora* mitogenomes against their nuclear genomes (NCBI: AEIT00000000.1; JGI: Project: 1063673).

### 2.4 Intron analysis

Introns in *cox1* genes of 76 published Basidiomycota mitogenomes were classified into different position classes (Pcls) according to the method described by Férandon et al. [43]. The *cox1* genes of 76 Basidiomycota mitogenomes were first aligned with the *cox1* gene of the medical fungus *Ganoderma calidophilum* [18] by Clustal W [44], which we used as the reference. Each Pcl was constituted by introns inserted at the same position of corresponding *cox1* gene and namely by the insert sites (nt) in the reference gene. The same Pcl from different species was considered as orthologous intron and usually has a high sequence similarity.

### 2.5 Phylogenetic analysis

In order to investigate the phylogenetic positions of the two *Coniophora* species and other Boletales species in the phylum Basidiomycota, we constructed a phylogenetic tree of 77 species based on the combined mitochondrial gene set (15 core PCGs + 2 rRNA genes) [29]. We used *Annulohypoxylon stygium* from the phylum Ascomycota as an outgroup [45]. Individual mitochondrial genes were first aligned using the MAFFT v7.037 [46], and then concatenated into a combined mitochondrial gene set using SequenceMatrix v1.7.8 [47]. Partition homogeneity test was used to detect potential phylogenetic conflicts between different mitochondrial genes. Best-fit models of phylogeny and partitioning schemes for the gene set were determined using PartitionFinder v2.1.1 [48]. We conducted the phylogenetic analysis using both bayesian inference (BI) and maximum likelihood (ML) methods [49]. MrBayes v3.2.6 [50] was used to perform the BI analysis, and RAxML v 8.0.0 [51] was used for the ML analysis.

### 2.6 Data availability

The complete mitogenomes of *C. olivacea* and *C. puteana* were deposited in the GenBank database under the accession number MT375015 and MT375016, respectively.

## 3 Results

### 3.1 Features and PCGs of *Coniophora* mitogenomes

**Fig. 1:**
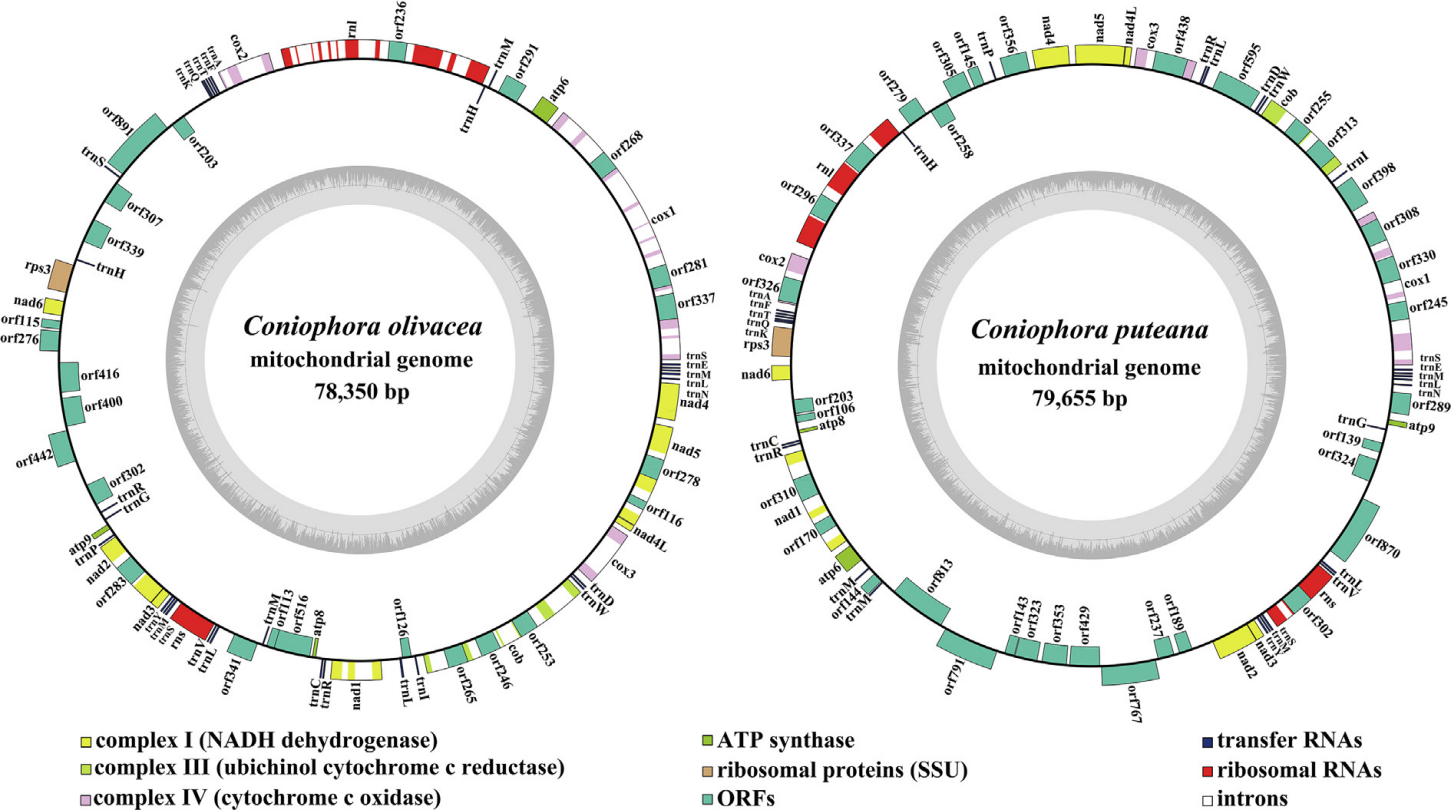
Circular maps of the two *Coniophora* mitogenomes. Genes are represented by different colored blocks. Colored blocks outside each ring indicate that the genes are on the direct strand, while colored blocks within the ring indicates that the genes are located on the reverse strand.

Both *C. olivacea* and *C. puteana* mitogenomes were circularly assembled, with the total sizes of 78,350 bp and 79,655 bp, respectively (Fig. 1). The GC contents of *C. olivacea* and *C. puteana* mitogenomes were 27.32% and 27.61%, respectively, with an average GC content of 27.47% (Table S1). Both the two species contained negative AT skews and positive GC skews. A whole set of core PCGs was detected in the *C. olivacea* and *C. puteana* mitogenomes, respectively, which included *atp6*, *atp8*, *atp9*, *cob*, *cox1*, *cox2*, *cox3*, *nad1*, *nad2*, *nad3*, *nad4*, *nad4L*, *nad5*, *nad6* and *rps3*. In addition, fifteen and twenty-three free-standing PCGs (non-intron encoding ORFs) were detected in *C. olivacea* and *C. puteana* mitogenomes, respectively. The two *Coniophora* mitogenomes both contained 7 plasmid-derived genes (genes encoding DNA polymerases). Additionally, the *C. olivacea* and *C. puteana* mitogenomes contained 8 and 16 PCGs with unknown functions, respectively. There were 33 and 13 introns detected in the *C. olivacea* and *C. puteana* mitogenomes, respectively, which harboured 10 and 12 intron encoding ORFs, respectively. Intron encoding ORFs in the two *Coniophora* mitogenomes mainly encoded GIY-YIG and LAGLIDADG endonucleases (Table S2). In the mitogenome of *C. olivacea*, ORFs in introns mainly encoded LAGLIDADG endonucleases, which were four times as many as GIY-YIG endonucleases. However, most of intron encoding ORFs in the *C. puteana* mitogenome encoded GIY-YIG endonucleases.

### 3.2 rRNA genes and tRNA genes

Two rRNA genes were detected in the two *Coniophora* mitogenomes, including the large subunit ribosomal RNA gene (*rnl*) and small subunit ribosomal RNA (*rns*) (Table S2). There were 10, 2, and 1 introns detected in *rnl* gene of *C. olivacea*, *rnl* and *rns* genes of *C. puteana*, respectively. The average sizes of *rnl* and *rns* genes in the two *Coniophora* mitogenomes were 3,150 and 1,629 bp, respectively. The *C. olivacea* mitogenome contained longer *rnl* and shorter *rns* genes than the *C. puteana* mitogenome.

**Fig. 2:**
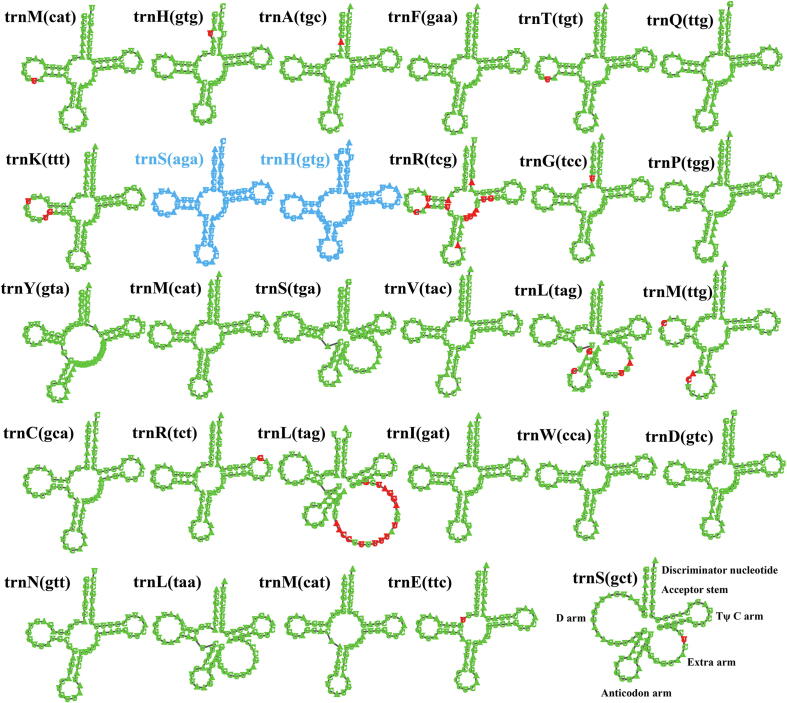
Putative secondary structures of tRNA genes in the two *Coniophora* mitogenomes. tRNA genes with green color represent tRNAs shared by the two *Coniophora* species, while tRNA genes with blue color represent the unique tRNAs in *Coniophora olivacea*. Residues conserved across the two mitogenomes are shown in green, while variable sites are shown in red. All genes are shown in order of occurrence in the mitogenome of *C. olivacea*, starting from *trnM*. (For interpretation of the references to color in this figure legend, the reader is referred to the web version of this article.)

Twenty-nine and twenty-seven tRNA genes were detected in the mitogenomes of *C. olivacea* and *C. puteana*, respectively, which encoded 20 standard amino acids (Fig. 2). The *C. puteana* mitogenome contained two additional *trnS* and *trnH* genes compared with the *C. puteana* mitogenome. All tRNAs in the two mitogenomes were folded into classical cloverleaf structures, with individual tRNA gene varied between 70 and 95 bp. The *trnL* gene in the *C. olivacea* mitogenome contained a large extra arm (25 bp), which resulted in the *trnL* gene the largest tRNA gene among all tRNAs in the two *Coniophora* mitogenomes. Of the 27 tRNAs shared in the two *Coniophora* mitogenomes, 13 contained sites that varied between the two mitogenomes. A total of 46 variable sites were detected in the 27 tRNA genes between the two species, of which 22 occurred on the extra arm, suggesting that the extra arm was highly variable in the two *Coniophora* mitogenomes.

### 3.3 Repetitive elements in *Coniophora* mitogenomes

By BlastN searches of the two *Coniophora* mitogenomes against themselves, we identified 32 and 27 intra-genomic duplications in the *C. olivacea* and *C. puteana* mitogenomes, respectively (Table S3). The length of these duplications in the two *Coniophora* mitogenomes ranged from 35 bp to 851 bp, with pair-wise nucleotide similarities ranging from 71.29% to 100%. The largest repeats were observed in the intergenic region between *trnM* and *orf813*, as well as in the protein coding region of *orf767* in the mitogenome of *C. puteana*. The largest repeats in the *C. olivacea* mitogenome were found located in the protein coding regions of *orf891* and *orf516*, with each repeating sequence 493 bp long. Repeat sequences accounted for 7.06% and 10.07% of the *C. olivacea* and *C. puteana* mitogenomes, repectively. Using Tandem repeat finder, we identified 6 and 15 tandem repeats in the *C. olivacea* and *C. puteana* mitogenomes, repectively (Table S4). The longest tandem repeat sequence was found located in the intergenic region between *orf339* and *trnH* from the *C. olivacea* mitogenome, with a length of 74 bp. Most of tandem repeats in the two *Coniophora* mitogenomes contained 2 copies. Tandem repeated sequences accounted for 0.40% and 0.80% of the *C. olivacea* and *C. puteana* mitogenomes, repectively.

To detect if there was any gene fragment that naturally transferred between the mitochondrial and nuclear genomes of the two *Coniophora* species, we BlastN searches of the two newly sequenced mitogenomes with their published nuclear genomes. A total of 4 and 31 repetitive fragments were identified between mitochondrial and nuclear genomes of *C. olivacea* and *C. puteana*, respectively (Table S5). These repetitive fragments ranged from 31 bp to 141 bp in length, with pair-wise nucleotide similarities ranging from 90.20% to 100%. The largest repeat fragment was found located in the intergenic region between *trnM* and *orf813* in the *C. puteana* mitogenome. A total of 193 bp and 1,973 bp aligned fragments were detected in the *C. olivacea* and *C. puteana* mitogenomes, respectively, indicating natural gene transfer events between mitochondrial and nuclear genomes may have occurred during the evolution process of *Coniophora* species.

### 3.4 Mitochondrial gene arrangement in Boletales species

**Fig. 3:**
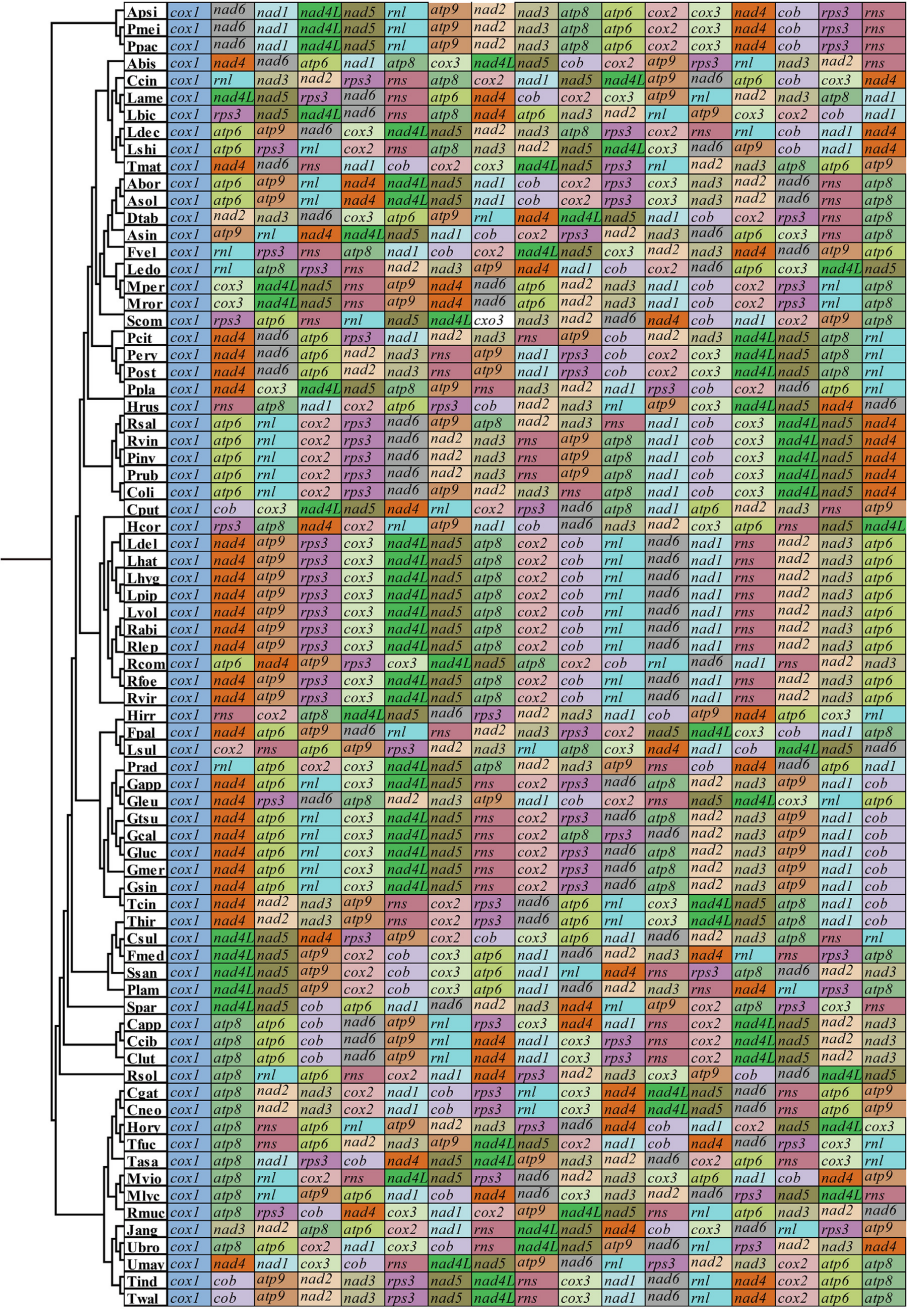
Mitochondrial gene arrangement analyses of 76 Basidiomycota species. All genes are shown in order of occurrence in the mitochondrial genome, starting from *cox1*. Fifteen core protein coding genes and two rRNA genes were included in the gene arrangement analysis. The phylogenetic positions of 76 Basidiomycota species were established using the Bayesian inference (BI) method and Maximum Likelihood (ML) method based on concatenated mitochondrial genes. Species and NCBI accession number used for gene arrangement analysis in the present study are listed in Supplementary Table S6.

In this study, the arrangements of 15 core PCGs and 2 rRNA genes in 76 Basidiomycota species were compared (Fig. 3). We found that the gene arrangement of Basidiomycetes varied greatly at the order level, and any fungus from different orders contained inconsistent gene arrangement. In the case of Boletales, it was found that there were three Boletales species with the same gene arrangement, including *R. vinicolor*, *P. involutus* and *P. rubicundulus*, which may represent the gene order of the common ancestor of Boletales. Compared with the putative gene arrangement of Boletales ancestor, the other three Boletales species underwent large-scale gene rearrangements, even between species from the same genus, such as *Coniophora*, and *Rhizopogon*. In *C. olivacea* and *R. salebrosus*, we observed gene position exchange within species compared with gene orders of other Boletales species. The results showed that the arrangement of mitochondrial genes in the two *Coniophora* species is highly variable.

**Fig. 4:**
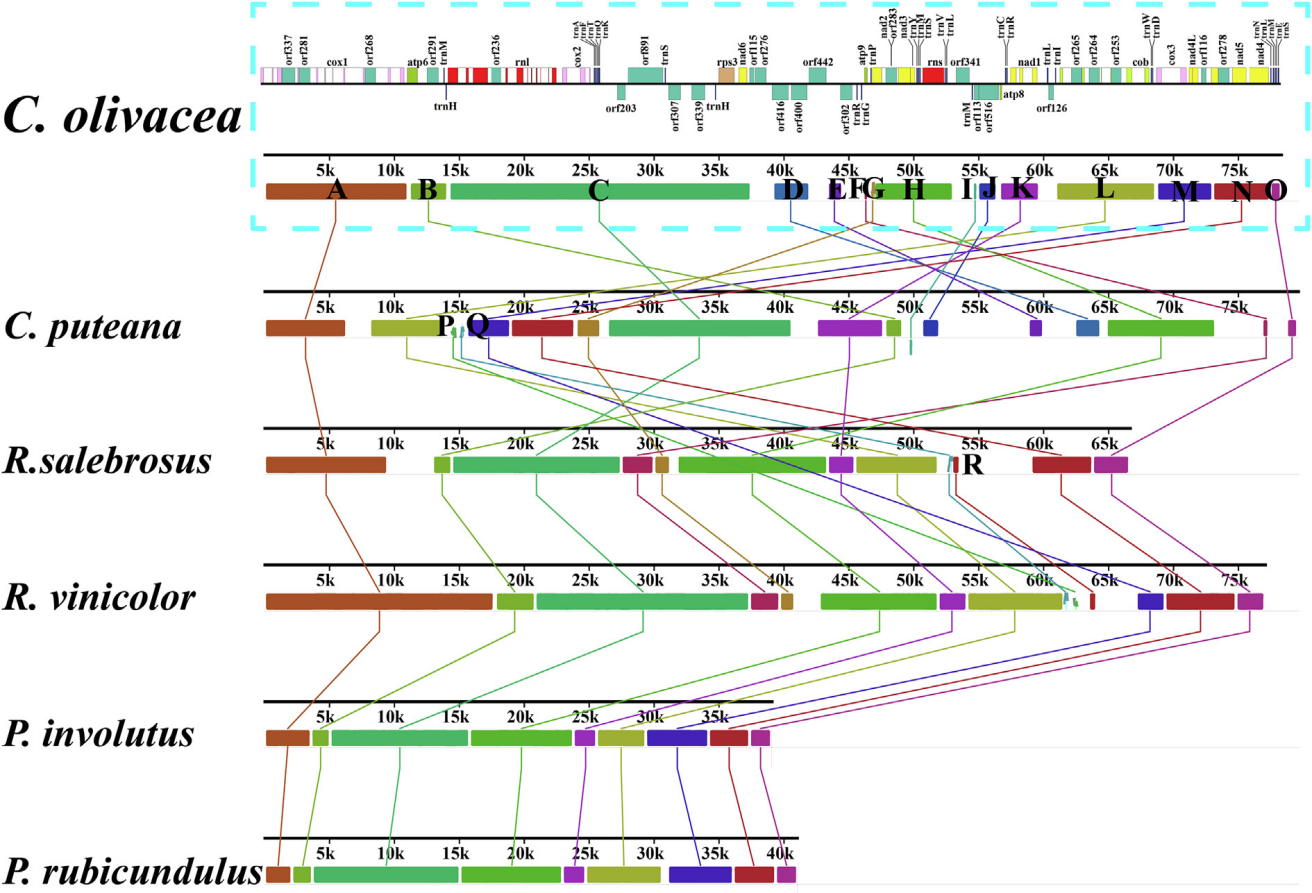
Gene collinearity analysis of 6 Boletales species using Mauve v2.4.0. Color blocks of the same color represent homologous regions between different mitogenomes. The schematic diagram of *Coniophora olivacea*'s mitogenome is shown at the top of the picture.

The complete mitogenomes of 6 Boletales species were analyzed by collinearity analysis, and 18 homologous regions were detected in the 6 Boletales species (Fig. 4). *Coniophora olivacea* was found had lost homologous regions P, Q and R, while *C. puteana* lacked homologous regions R. Homologous regions D, E, I, and J were unique homologous regions in *Coniophora* species, which have never been detected in the other Boletales species. These unique homologous regions were related to *orf416*, *orf400* and *orf302* encoding proteins with unknown functions and *orf113* and *orf516* encoding DNA polymerases. Collinearity analysis showed that *Rhizopogon* species and *Paxillus* species had a high degree of collinearity within and between genera, while *Coniophora* species showed large-scale gene rearrangements, indicating variability of gene arrangement and gene content in *Coniophora* species.

### 3.5 Genetic distance, and evolutionary rates of core PCGs

**Fig. 5:**
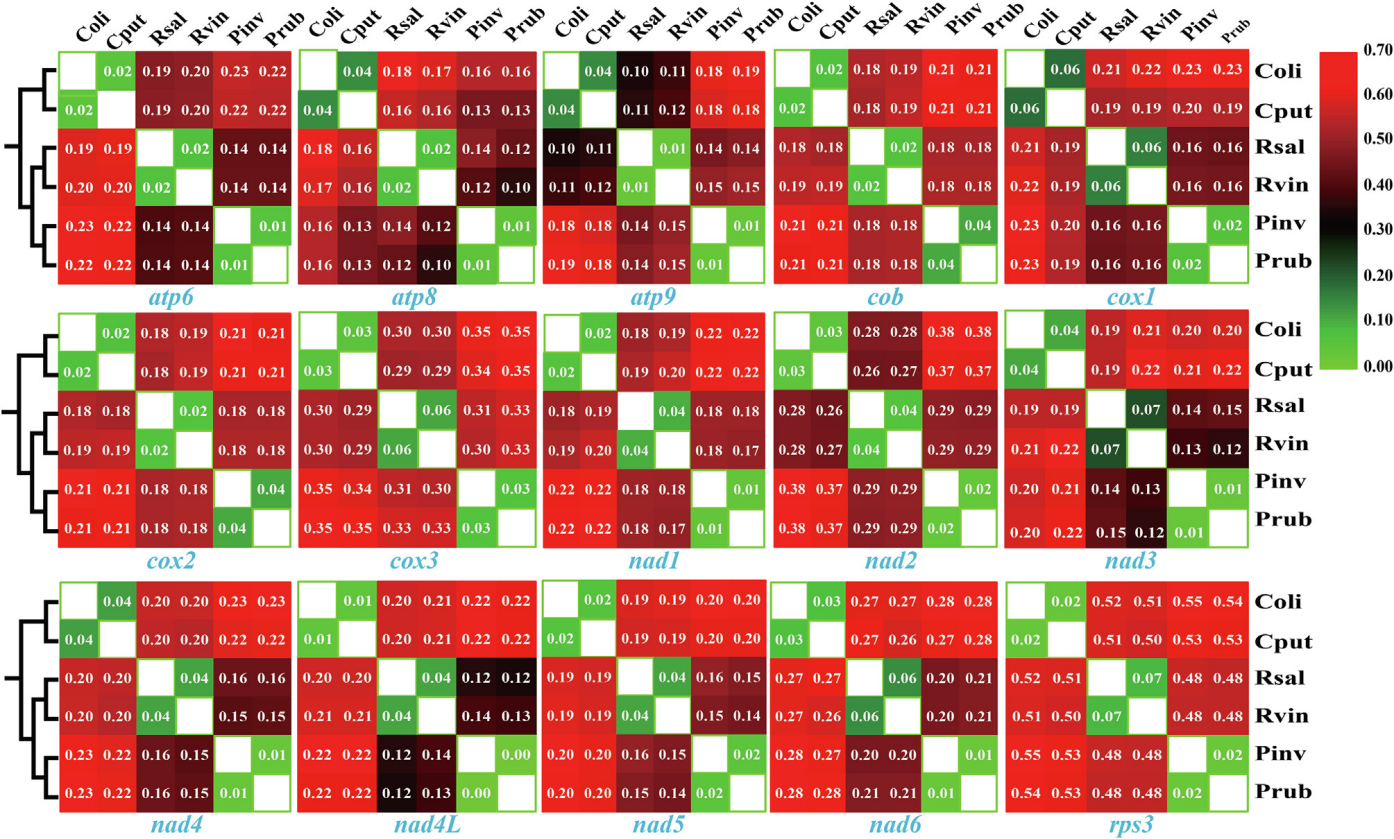
Pairwise genetic distances between each pair of the 15 core PCGs in the 6 Boletales mitogenomes based on the Kimura-2-parameter model. Species and NCBI accession number used for genetic distance analysis in the present study are listed in Supplementary Table S6.

The *rps3* gene was found had the largest K2P genetic distance between the 6 Boletales species (overall mean 0.41), which indicted this gene had the fastest mutation rate among the 15 core PCGs in Boletales (Fig. 5). The *cox3* and *nad6* genes also showed great gene differentiations between the 6 Boletales species, with an overall mean K2P distance of 0.26 and 0.21, respectively. The *atp8* and *atp9* genes had the lowest mean K2P genetic distance between the 6 Boletales species, indicating that the two genes were highly conservative. We found that there was a close genetic distance between species within the same genera, while the K2P genetic distances between species from different genera varied greatly.

**Fig. 6:**
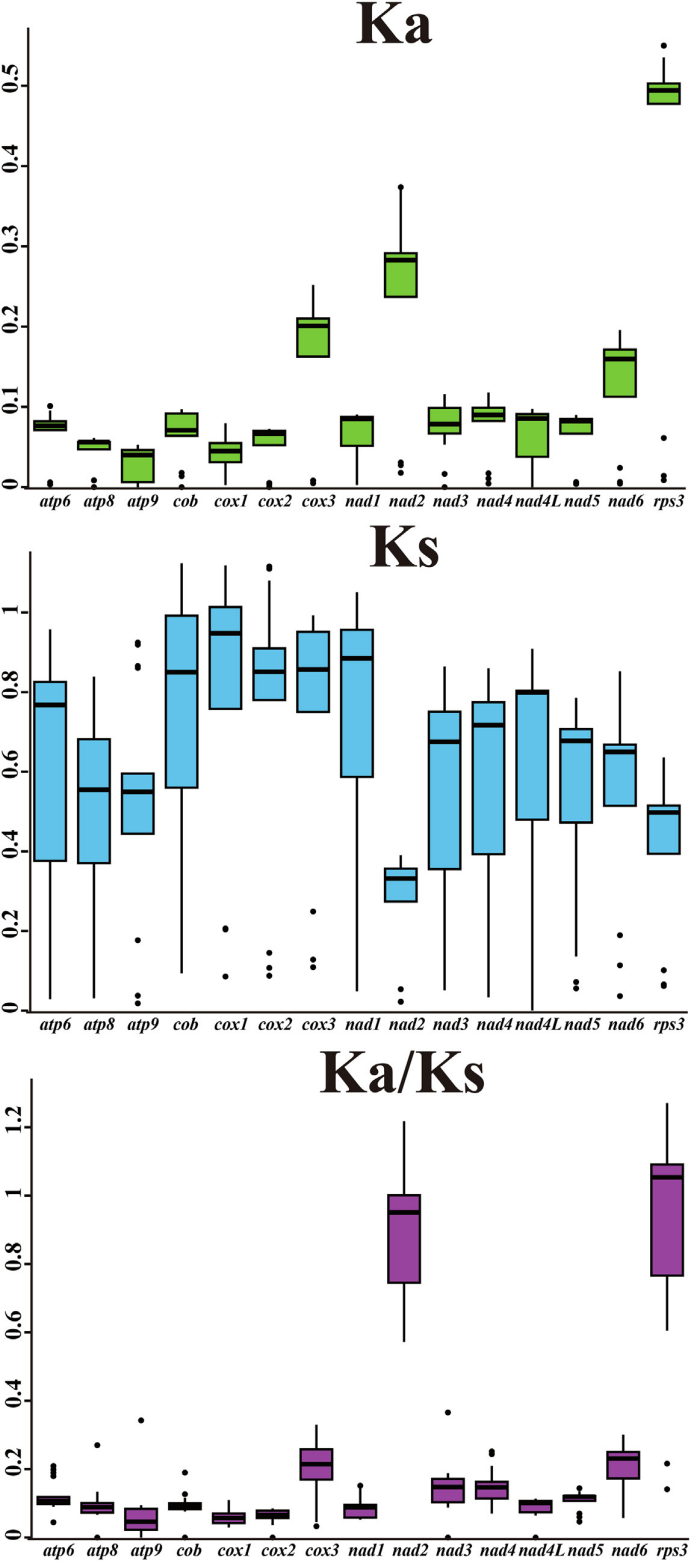
Genetic analysis of 15 core protein coding genes conserved in the 6 Boletales mitogenomes. *Ka*, nonsynonymous substitution site; *Ks*, synonymous substitution site.

The *rps3* gene was detected had the highest mean non-synonymous substitution rate (*Ka*) among the 15 core PCGs detected (average value 0.41), followed by the *nad3* gene (average value 0.08) (Fig. 6). While the *atp9* gene had the lowest mean *Ka* value among the 15 core PCGs from the 6 Boletales species (average value 0.03). The *cox1* gene was found exhibited the highest mean synonymous substitution rate (*Ks*) (average value 0.80), while *nad2* exhibited the lowest mean *Ks* value among the 15 PCGs detected (average value 0.27). The *Ka/Ks* values for 13 of the 15 core PCGs ranged from 0 to 0.37 (*Ka/Ks* < 1), indicating that these genes had been subjected to purifying selection in the process of evolution. However, the *Ka/Ks* values of the *nad2* and *rps3* genes were observed more than 1 between some species, including between *C. olivacea* and *C. puteana***,** between *R. vinicolor*, *P. involutus*, as well as between *R. vinicolor* and *P. rubicundulus*, indicating that the two genes may be under pressure of positive selection in some Boletales species.

### 3.6 Intron dynamics of *cox1* gene in Basidiomycota species

Introns (coding introns or noncoding introns) could be classified into different Pcls according to their insertion sites in the protein coding region of host gene. Introns belonging to the same Pcls were considered to be homologous and showed high sequence similarities. In the present study, we analyzed the intron dynamics of 76 Basidiomycota mitogenomes, which accounted for 2/3 of Basidiomycota mitogenomes available in the NCBI database. A total of 1058 introns were detected in the 76 Basidiomycota species, with each species containing 0 to 46 introns. Introns were unevenly distributed in core PCGs and rRNA genes of Basidiomycota species. It was found that the *cox1* gene was the largest host gene of introns in Basidiomycota, and about 45.46% of introns were located in it.

**Fig. 7:**
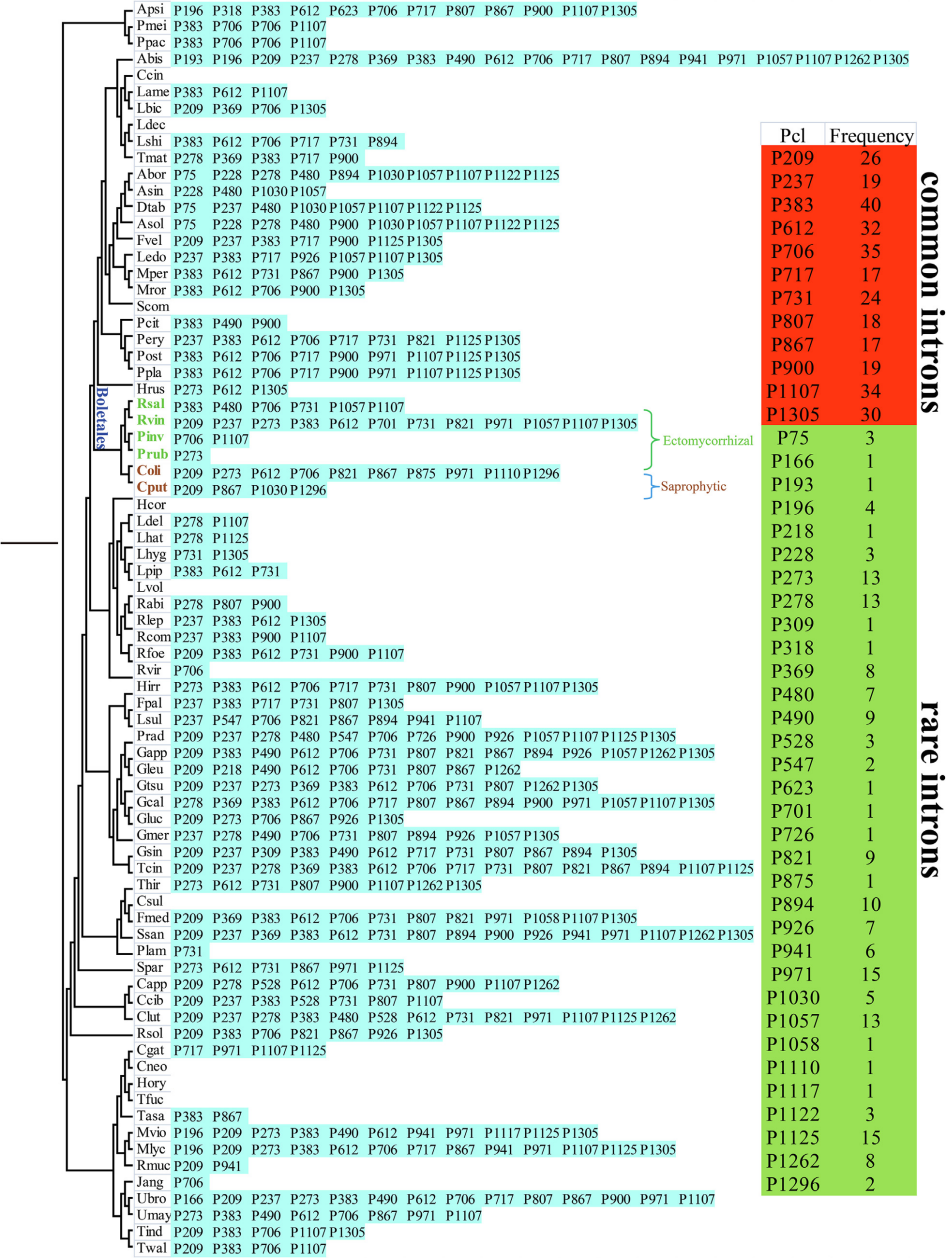
Position class (Pcl) information of *cox1* gene in the 76 Basidiomycota species. Introns in *cox1* genes of 76 published Basidiomycota mitogenomes were classified into different position classes (Pcls) using the cox1 gene of medical fungus *Ganoderma calidophilum* as the reference. Each Pcl was constituted by introns inserted at the same position of corresponding *cox1* gene and named according to its insertion site in the aligned corresponding reference sequence (nt). The Pcls present in more than 1/5 of Basidiomycota species were considered as common Pcls in Basidiomycota, while introns detected in less than 1/5 of Basidiomycota species were considered to be rare introns. The phylogenetic positions of 76 Basidiomycota species were established using the Bayesian inference (BI) method and Maximum Likelihood (ML) method based on concatenated mitochondrial genes. Species information is shown in Supplementary Table S6.

We further studied the dynamic changes of introns in *cox1* genes of Basidiomycota. The *cox1* gene of medical fungus *G. calidophilum* [18] was used as the reference to determine corresponding insertion sites of introns in Basidiomycota species. A total of 45 Pcls were detected in *cox1* genes of 76 Basidiomycota species (Fig. 7). *Agaricus bisporus* [52] was found containing the largest number of Pcls, while *cox1* genes of species, such as *Coprinopsis cinerea* [53], *Lyophyllum decastes* [54], and *Schizophyllum commune* [55] did not contain any introns. Pcls present in more than 1/5 of Basidiomycota species were considered as common Pcls in Basidiomycota, while introns detected in less than 1/5 of Basidiomycota species were considered to be rare introns. In this study, 12 Pcls were detected widely distributed in Basidiomycota species, of which P383 was the most common, which distributed in 40 of the 76 Basidiomycota species, followed by the P706, which could be detected in 35 Basidiomycota species. Pcls, including P166, P193, and P218, could only be detected in one of 76 Basidiomycota species. The 6 Boletales species were found containing 19 Pcls, 9 of which were common introns in Basidiomycota, including P209, P383, P612, etc. The class and quantity of introns in different Boletales species varied greatly, which indicated that the loss/gain of introns occurred in the evolution of Boletales. The two newly sequenced Boletales species contained a Pcl (P867), which was not found in other Boletales but in distant species, such as *Laetiporus sulphureus* [29] and *Ustilago maydis*, indicating potential intron transfer events. In addition, the two *Coniophora* species also contained several novel introns in Basidiomycota, including P875, P1110 and P1296, and none of these unique introns has been detected in Boletales and Basidiomycota.

### 3.7 Comparative mitogenomic analysis and phylogenetic analysis

Comparative mitogenomic analysis showed that the two *Coniophora* mitogenomes were the largest among the 6 Boletales mitogenomes tested, indicating that the *Coniophora* mitogenomes experienced mitogenome expansions in the evolutionary process (Table S1). The GC content of the two *Coniophora* mitogenomes (average 27.47%) was higher than the GC contents of other Boletales species (21.42%). All the 6 Boletales species contained negative AT skews and positive GC skews. Comparative analysis of gene content indicated that the two *Coniophora* species contained the most number of non-intron encoding PCGs among the 6 Boletales species, which was mainly due to the expansion of plasmid-derived genes (genes encoding DNA polymerases) and unknown functional genes in the two saprophytic Boletales species. *C. olivacea* was found containing the most introns (33) in Boletales species. However, only 30.30% introns in *C. olivacea* contained intron encoding ORFs, while 92.31% introns in *C. puteana* contained intron encoding ORFs. The results indicated that a large number of introns in *C. olivacea* lost their homing ORFs. The two *Coniophora* species also contained the most number of tRNA genes among the 6 Boletales reported. We found that the intra-genomic repeats accumulated in the two saprophytic Boletales species (the genus *Coniophora*) relative to the four ectomycorrhizal Boletales species we tested, and the two saprophytic Boletales species also contained higher protein coding regions due to accumulation of plasmid-derived genes and non-conserved genes.

**Fig. 8:**
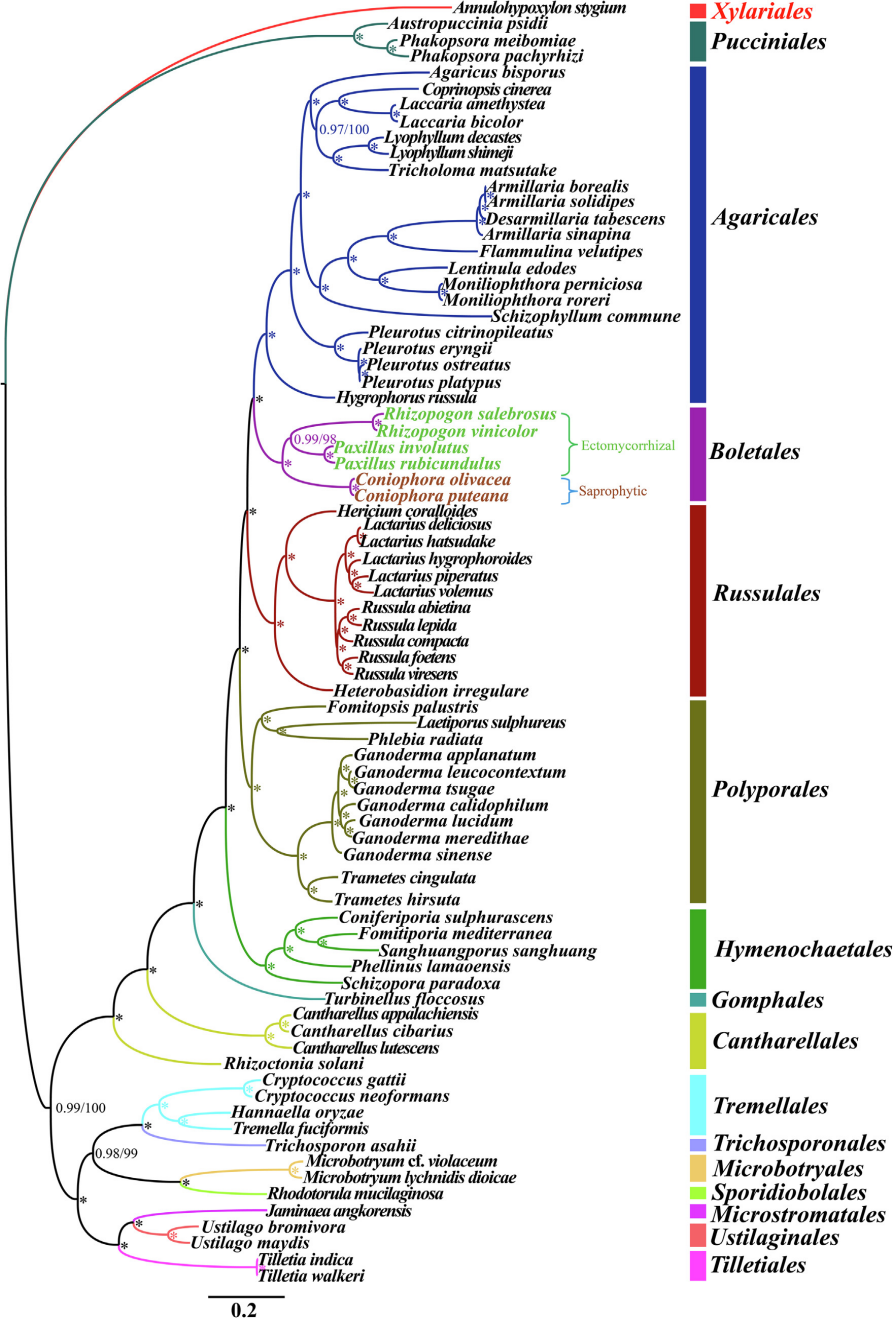
Molecular phylogeny of 76 Basidiomycota species based on Bayesian inference (BI) and Maximum likelihood (ML) analysis of 15 protein coding genes and two rRNA genes. Support values are Bayesian posterior probabilities (before slash) and bootstrap (BS) values (after slash). The asterisk indicates that the BPP value is 1 and the BS value is 100 of the branch. Species and NCBI accession numbers for mitogenomes used in the phylogenetic analysis are provided in Supplementary Table S6.

We obtained identical tree topologies using both Bayesian inference (BI) and Maximum likelihood (ML) methods based on the combined mitochondrial gene set (15 core PCGs + 2 rRNA genes) (Fig. 8). All major clades within the trees were well supported (BPP ≥ 0.97; BS ≥ 98). Based on the phylogenetic analyses, the 76 *Basidiomycota* species could be divided into 14 major clades, corresponding to the orders Pucciniales, Agaricales, Boletales, Russulales, Polyporales, Hymenochaetales, Cantharellales, Tremellales, Trichosporonales, Microbotryales, Sporidiobolales, Microstromatales, Ustilaginales, and Tilletiales (Table S6). The 6 Boletales species could be divided into two groups, wherein the first comprised two species form the *Coniophora* genus, and the second group comprised four species within the *Paxillus* genus and *Rhizopogon* genus. The results indicated that *Coniophora* differentiated from Boletales in the early stage .

## 4 Discussion

### 4.1 Gene content variation in Boletales mitogenomes

In the present study, we found the core PCGs [56], [57] underwent non synonymous and synonymous mutations in Boletales, and different core PCGs showed differentiated mutation rate. The results were consistent with previous studies [58], [59]. The *rps3* gene was found differentiated greatly in Boletales species. In addition, two core PCGs, including *nad2* and *rps3*, were observed subjected to strong pressure of positive selection between some Boletales species (Ka/Ks > 1). Different Boletales species have varied life styles, different host plant preferences and diverse habitat environments [5], [60]. The positive selection pressure on *nad2* and *rps3* genes may result from differentiations of life styles and environmental adaptations.

In addition to these core PCGs, non-conserved PCGs were frequently detected in mitogenomes of Basidiomycota, including intron encoding ORFs, plasmid-derived genes and ORFs with unknown functions. We found that there were more plasmid-derived genes and non-conserved ORFs in the two saprophytic Boletales species than in the four ectomycorrhizal Boletales species. Plasmid-derived genes were thought to be obtained from mitochondrial plasmids [61], which were considered as self-replicating genetic elements. Mitochondrial plasmids were commonly observed in the mitogenomes of plants and fungi and were believed to have a separate evolutionary history from their hosts [62], [63]. In some Basidiomycota species, mitochondrial plasmids were free-living [64], [65], while in other species, they were integrated into the mitogenome of Basidiomycota [63], [66]. The impact of its dynamic changes on the evolution and function of mitogenome is still unknown. In addition, more non-conserved ORFs were detected in the two *Coniophora* mitogenome than the other ectomycorrhizal Boletales species, and their functions need to be further revealed to promote a comprehensive understanding of mitochondrial functions and evolution in saprophytic Boletales species. Compared with the four ectomycorrhizal Boletales species, the saprophytic Boletales species contained more tRNA genes. In addition, we found that the tRNA of *Coniophora* species underwent base mutation. Previous studies have found that mutations in tRNAs could affect protein synthesis and metabolism of eukaryotes [67], [68]. The effect of tRNA mutation on the growth and development of *Coniophora* species needs to be further investigated. In general, the variations of gene contents, including intron encoding ORFs, plasmid-derived gene, non-conserved PCGs and tRNA genes, led to variable *Coniophora* mitogenomes.

### 4.2 Introns dynamics in Boletales and Basidiomycota mitogenomes

Introns are considered to be one of the main factors leading to the size variations of mitogenomes in fungi [52], [69], [70]. Compared with the mitochondrial introns of plants, most of which belongs to the group II, while most fungal introns are found belonging to the group I [71]. In the present study, 1058 introns were detected in 76 Basidiomycota species, with an average of 14 introns in each Basidiomycota species. These introns were unevenly distributed in species and genes of the phylum Basidiomycota, and the *cox1* gene was the largest host gene of Basidiomycete introns. The classes of Basidiomycete introns in *cox1* genes were diverse, and a total of 45 Pcls were detected in *cox1* genes of Basidiomycota species. We found that the type and number of introns in *cox1* gene of the 6 Boletales species varied greatly, which indicated that intron loss/gain may have occurred in the evolution of Boletales. Some intron types were found widely distributed in Boletales species, and they may be obtained from the common ancestor of Boletales. In addition, several Pcls could only be detected in one of the 6 Boletales species, and homologous introns were detected in distant species, indicating potential intron transfer events [52]. Interestingly, we found several novel Pcls in two saprophytic Boletales species, which were not detected in other Boletales species and Basidiomycota species. The origin and evolution of these novel Pcls need to be further investigated, which will help us to understand the evolution of the order Boletales. In addition, we found that the *C. olivacea* with the most introns among the 6 Boletales species lost most of the intron encoding ORFs, and the *P. rubicundulus* species lost all of the intron encoding ORFs, indicating that the introns of Boletales are undergoing contraction.

### 4.3 Mitochondrial gene rearrangements in Boletales species

The rearrangement of mitochondrial genes is one of the important references to reflect the evolutionary status of eukaryotes [22], [72], [73]. In the past, the arrangement of mitochondrial genes in animals was considered to be conservative. However, with more and more animal mitogenomes having been obtained, gene rearrangements have been frequently observed in animals, and several models have been proposed to reveal the rearrangement of animal mitogenome [74], [75]. Compared with the arrangement of animal mitogenomes, the arrangement of fungal mitogenome is highly variable. However, no model has been proposed to reveal the mechanism of mitogenome rearrangement in fungi. In this study, we found that three ectomycorrhizal Boletales species, including *R. vinicolor* [9], *P. involutus*, and *P. rubicundulus*, had identical gene arrangement, which may represent the gene arrangement of the common ancestor of the 6 Boletales species. Compared with the mitochondrial gene arrangement of the presumed ancestor of the 6 Boletales, large-scale gene rearrangements were observed in the two saprophytic Boletales species (*Coniophora*) and one ectomycorrhizal Boletales species (*R. salebrosus*), including gene position exchanges, possible gene migrations and gene insertions. Previous studies have shown that the accumulation of repetitive sequences may lead to the recombination of fungal mitogenome, and thus contributing to the rearrangement of fungal mitogenome [71]. In the present study, we observed a large amount of repeat sequences in two saprophytic Boletales species, which may be one of the main factors contributing to large-scale gene rearrangements in the two *Coniophora* species. However, in *R. salebrosus* mitogenome [9], no intra-genomic duplications has been detected, but gene rearrangements have been detected, indicating that there may be other driving mechanisms for mitogenome rearrangement of *Rhizopogon* species, which needs further study.

### 4.4 Phylogeny of Basidiomycota based on mitochondrial genes

The phylum Basidiomycota is the largest group of mushroom-forming fungi on the earth, which plays an important role in the natural cycle, ecological protection and medical or industrial application [17], [62], [76]. Understanding the origin and evolution of Basidiomycetes contributes to the understanding and utilization of Basidiomycetes in human life. However, it is very difficult to classify Basidiomycetes only by morphology even in today's rapid development of microscopic observation equipment, because many Basidiomycetes have variable and overlapping morphological characteristics [54]. Therefore, the introduction of molecular markers can greatly promote the understanding of the inheritance, origin and evolution of Basidiomycetes [77]. Up to now, rRNA internal transcribed spacer (ITS) sequence has been widely used in the phylogeny of Basidiomycetes and other fungi [78]. Nuclear genome has also become an important option in the phylogeny of Basidiomycetes, because it can provide enough evolutionary information [79], [80]. Compared with ITS sequence and nuclear genomes, mitogenome provide adequate genetic information and is easy to obtain, thus becoming an important tool to understand the origin, classification and evolution of Basidiomycetes [20], [81], [82], [83]. However, compared with the available nuclear genome of Basidiomycetes, the number of complete mitogenome of Basidiomycetes is far from enough (https://www.ncbi.nlm.nih.gov/genome/browse#!/overview/), which limits the large-scale application of this important molecular marker in phylogeny of Basidiomycetes. In this study, 2/3 of the complete mitogenomes of Basidiomycetes available in NCBI were included in the phylogeny study. Based on ML and BI methods, we obtained well-supported phylogenetic tree for 76 Basidiomycota species, which showed that mitochondrial gene was a powerful tool to analyze the phylogenetic relationships of Basidiomycetes. In addition, based on the phylogenetic tree, we found that the *Coniophora* species differentiated from the order Boletales in the early stage. More mitogenomes of Boletales species need to be obtained to understand the origin and evolution patterns of Boletales species with complex lifestyles.

## Funding

This study was supported by the project of protection, breeding and high value utilization of Plateau Characteristic edible fungi resources (2019ZG00906), the project of analysis of species diversity and ecological adaptation mechanism of Boletales based on mitochondrial genome (2019ZG00909-04) and the high tech field expansion project of Sichuan Academy of Agricultural Sciences (2018GXTZ-001).

## 6 Contributions

Data curation and Formal analysis: Q.L., C.X., Z.B., L.L., and W.T.. Funding acquisition and Project administration: P.L., W.H., and M.G.. Visualization: X.J.. Writing - original draft: Q.L. Writing - review & editing: P.W., and Q.L.

## Declaration of Competing Interest

The authors declare that they have no known competing financial interests or personal relationships that could have appeared to influence the work reported in this paper.
